# HCT-Det: A High-Accuracy End-to-End Model for Steel Defect Detection Based on Hierarchical CNN–Transformer Features

**DOI:** 10.3390/s25051333

**Published:** 2025-02-21

**Authors:** Xiyin Chen, Xiaohu Zhang, Yonghua Shi, Junjie Pang

**Affiliations:** 1 School of Mechanical and Automotive Engineering, South China University of Technology, Guangzhou 510640, China; 201910100254@mail.scut.edu.cn (X.C.); 202320100560@mail.scut.edu.cn (J.P.); 2 Hymson Laser Technology Group Co., Ltd., Shenzhen 518110, China; zhangxiaohujack@hymson.com

**Keywords:** transformer, convolutional neural network, defect detection

## Abstract

Surface defect detection is essential for ensuring the quality and safety of steel products. While Transformer-based methods have achieved state-of-the-art performance, they face several limitations, including high computational costs due to the quadratic complexity of the attention mechanism, inadequate detection accuracy for small-scale defects due to substantial downsampling, inconsistencies between classification scores and localization confidence, and feature resolution loss caused by simple upsampling and downsampling strategies. To address these challenges, we propose the HCT-Det model, which incorporates a window-based self-attention residual (WSA-R) block structure. This structure combines window-based self-attention (WSA) blocks to reduce computational overhead and parallel residual convolutional (Res) blocks to enhance local feature continuity. The model’s backbone generates three cross-scale features as encoder inputs, which undergo Intra-Scale Feature Interaction (ISFI) and Cross-Scale Feature Interaction (CSFI) to improve detection accuracy for targets of various sizes. A Soft IoU-Aware mechanism ensures alignment between classification scores and intersection-over-union (IoU) metrics during training. Additionally, Hybrid Downsampling (HDownsample) and Hybrid Upsampling (HUpsample) modules minimize feature degradation. Our experiments demonstrate that HCT-Det achieved a mean average precision (mAP@0.5) of 0.795 on the NEU-DET dataset and 0.733 on the GC10-DET dataset, outperforming other state-of-the-art approaches. These results highlight the model’s effectiveness in improving computational efficiency and detection accuracy for steel surface defect detection.

## 1. Introduction

Steel is of paramount importance for the national economy and everyday life. During production, processing, and usage, surface defects such as cracks, holes, inclusions, and wrinkles may arise in steel materials, affecting both the visual quality of the products and posing substantial risks for structural failure, which could lead to serious safety incidents and economic repercussions. Traditional manual visual inspection methods have failed to meet the rigorous standards demanded by modern industrial production due to their inherent inefficiencies, susceptibility to human error, and potential inaccuracies [1,2]. Therefore, developing accurate and reliable steel surface defect detection technology is crucial.

In the steel-manufacturing industry, automated surface defect detection has emerged as a critical research area. Traditional approaches have relied heavily on hand-crafted features and typically involve multiple steps, including image preprocessing, region-of-interest (RoI) extraction, feature extraction, feature selection, and pattern recognition. Leo et al. [3] developed an automated vision-based system for welding inspection for steel kegs, utilizing dual cameras, Canny edge detection, Radon transform, Scale-Invariant Feature Transform (SIFT) alignment, morphological filtering, and LBP-based template matching to detect defects with industrial robustness. Liu et al. [4] utilized local similarity analysis and neighborhood assessment to suppress noise, building models based on the statistical characteristics of image patches to identify defects. Suvdaa et al. [5] extracted features based on Scale-Invariant Feature Transform (SIFT), reduced dimensionality using principal component analysis, and applied Support Vector Machines (SVMs) for defect recognition. Luo et al. [6] employed generalized completed local binary patterns to facilitate rapid and accurate defect identification through mixed pattern encoding and histogram matching. Despite these contributions, traditional methods have been limited in their ability to capture deep-level information, especially in complex backgrounds and for varying defect morphologies, thereby restricting their generalizability and precision.

Recent advancements in deep learning within the computer vision domain have substantially propelled defect detection technology, allowing for the automatic extraction of high-level features. For instance, Wang et al. [7] proposed an integrated multi-level feature, Faster R-CNN, to address the variability and randomness of defects in metal plates. Dai et al. [8] improved upon Faster R-CNN to enhance precision in workpiece surface defect detection, while Yu et al. [9] introduced the SD-Net method, which integrates YOLOv3 with ResNet and an improved spatial pyramid module, thereby enhancing defect detection efficiency.

The Transformer architecture, which has garnered considerable attention for its global-feature-capturing capability, has demonstrated effectiveness in image classification and object detection, often outperforming convolutional neural networks (CNNs). Vision Transformer (ViT) [10], with its self-attention mechanism, effectively mitigates issues related to object size variation and position ambiguity. Fang et al. [11] utilized ViT to accurately detect steel surface defects using image patching and multi-head self-attention modules. Luo et al. [12] extended this approach by introducing CAT-EDNet, integrating cross-attention Transformers and refinement modules, thereby improving multi-scale feature optimization and bridging semantic gaps for more accurate boundary detection.

Nonetheless, Transformer-based defect detection models still suffer from several prominent shortcomings.

- High computational cost: The Transformer’s self-attention mechanism has a computational complexity that scales quadratically with the number of pixels, especially when processing high-resolution images. This results in significant computational overhead when handling large, detail-rich images, which hinders real-time processing capabilities and reduces efficiency.
- Limited precision for small-target detection: Although Transformers excel at capturing the global structures of images, they tend to underperform in detecting small targets due to the diffuse nature of their attention mechanisms. Small-target details, especially in complex backgrounds, may be overlooked, resulting in decreased detection precision for these objects.
- Inconsistency between classification and localization: Transformer models often face inconsistency between classification scores and IoU-based localization confidence, leading to erroneous detection outcomes. Bounding boxes may achieve high classification scores but low IoU (Intersection over Union) values, or vice versa, exacerbating false positives and missed detections, particularly in crowded or overlapping scenarios.
- Feature loss due to sampling methods: Standard upsampling and downsampling techniques often lead to feature degradation, particularly when employing methods like nearest-neighbor interpolation, deconvolution, max pooling, or convolutional downsampling. This resolution loss is detrimental, especially in regions with high-frequency details or ambiguous boundaries, impairing the model’s ability to accurately detect and localize defects.

To overcome these challenges, we propose a window-based self-attention (WSA) mechanism to minimize computational requirements while retaining crucial features. A parallel residual convolutional block (Res Block) further reinforces local feature connections, counteracting the potential loss from window-based constraints. The resulting backbone architecture, comprising WSA and Res Blocks, produces three cross-scale features, which are subsequently used as inputs for the encoder. The encoder employs Intra-Scale Feature Interaction (ISFI) to amplify the recognition of small targets, followed by Cross-Scale Feature Interaction (CSFI) to ensure comprehensive feature fusion and meet the detection requirements for targets of varying sizes. Moreover, we introduce a Soft IoU-Aware module during training to enforce a soft constraint, promoting alignment between high IoU and high classification scores. The incorporation of the composite HDownsample and HUpsample modules further mitigates feature loss during downsampling and upsampling operations. The proposed HCT-Det model consists of a backbone formed from WSA and Res Blocks, an encoder based on ISFI and CSFI, and a decoder employing Soft IoU-Aware operations, coupled with HDownsample and HUpsample modules for consistent feature quality. Experimental evaluations were performed to validate the model’s efficacy.

The remainder of this paper is organized as follows. Section 2 describes the architecture of HCT-Det in detail, elaborating on the WSA and Res Blocks, ISFI, CSFI, Soft IoU-Aware mechanism, and the HUpsample and HDownsample components. Section 3 presents experimental details, including dataset descriptions and performance analysis. Section 4 concludes the paper.

## 2. Methods

### 2.1. Architecture

**Figure 1:**
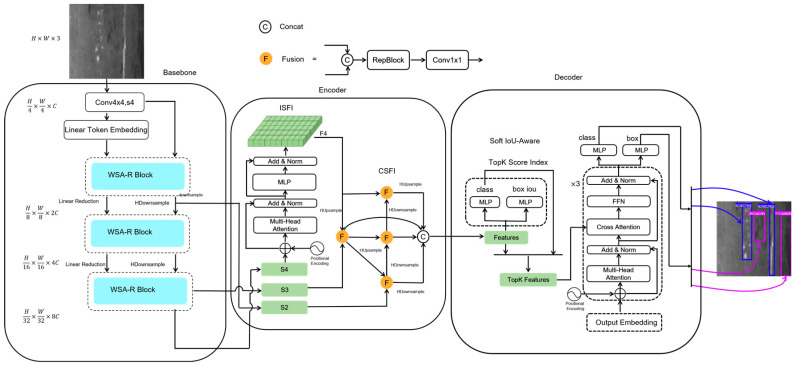
Architecture of the HCT-Det Model for steel defect detection.

The HCT-Det model consists of a backbone comprising combined convolutional neural network (CNN) and Transformer blocks, an encoder for multi-scale feature fusion, and a Transformer decoder that aids in auxiliary predictions. An overview of HCT-Det is given in Figure 1. Specifically, WSA Blocks and Res Blocks are utilized to extract both global and local features, and the features from the last three stages are input into the encoder as {S2, S3, S4}. The encoder first processes S4, using ISFI to derive F4, which is then fused across scales using CSFI based on S2, S3, and F4. The Soft IoU-Aware technique is employed to select a fixed number of encoder features as the initial object queries for the decoder. Subsequently, the decoder, which is equipped with an auxiliary prediction head, undergoes three iterations of the Transformer decoder to refine and generate the class and bounding box predictions.

### 2.2. WSA-R Fusion Block

The WSA-R fusion block is designed to address the computational inefficiencies of the original Transformer architecture, which employs a Multi-Head Self-Attention (MSA) mechanism. The MSA has a computational complexity that scales quadratically with the number of patches, making it impractical for high-resolution images. The complexity of MSA can be represented as

(1) $$\Omega \left(MSA\right)=4HWC^{2}+2(HW{)}^{2}C,$$

where H and W are the height and width of the feature map in patches, and C is the number of channels. This quadratic growth in complexity is a significant limitation for real-time processing and large-scale applications.

To mitigate this, we introduce the window-based self-attention (WSA) mechanism, which reduces computational complexity by dividing the feature map into non-overlapping windows. Each window is treated as a set of individual tokens, and self-attention is computed independently within each window. The complexity of WSA is significantly lower and can be represented as

(2) $$\Omega (WSA)=4HWC^{2}+2M^{2}HWC,$$

where M is the size of each window. By keeping M fixed (typically being set to 7), the complexity becomes linear with respect to the number of patches, making it more scalable for large feature maps.

As shown in Table 1, the WSA mechanism significantly reduces computational complexity compared to MSA, making it more suitable for high-resolution images and large feature maps. In WSA, self-attention is computed independently within each window, meaning that each window’s pixels (tokens) only interact internally, without considering neighboring windows. This approach significantly reduces computational complexity but sacrifices the global dependency between tokens.

To compensate for the loss of global dependency, we incorporate a residual convolutional block (Res Block). The Res Block includes the following features:

- A depthwise-separable convolution with a 7 × 7 receptive field, followed by Batch Normalization and ReLU activation.
- A 1 × 1 convolution in order to expand the channel dimension by a factor of four, followed by another Batch Normalization and ReLU layer.
- A 3 × 3 convolution in order to reduce the channel dimensions back to the original value.

The output of this process is then added to the input via a residual connection, enhancing local feature continuity and addressing potential losses due to window limitations.

**Figure 2:**
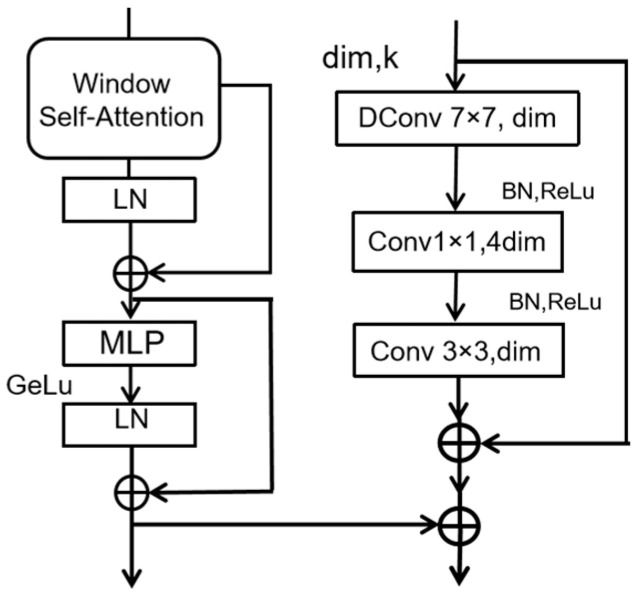
WSA-R Fusion Block.

The combination of the WSA Block and Res Block forms the WSA-R Fusion Block, which serves as the backbone of our model. This architecture effectively reduces the computational load associated with the Transformer while capturing both global and local details. The WSA-R Fusion Block is illustrated in Figure 2, showing how it integrates the benefits of both Transformer and CNN architectures to improve feature extraction and reduce computational demands.

### 2.3. ISFI and CSFI

In object detection tasks, small objects often present significant challenges due to their diminutive size and lack of distinctive features. To address this, we introduce two techniques: Internal-Scale Fusion (ISFI) and Cross-Scale Feature Integration (CSFI).

ISFI employs a Transformer encoder module, leveraging its multi-head self-attention, residual connections, and feed-forward neural networks to effectively extract and fuse sequence features from the deep S4 layer. This process not only enhances the model’s ability to capture the internal details of small targets but also enables it to discern complex inter-feature relationships through the self-attention mechanism, thereby improving small-object detection accuracy.

CSFI builds upon the concept of the Path Aggregation Network (PANet), which itself is an improvement over Feature Pyramid Networks (FPNs). PANet efficiently fuses multi-scale features through its repeated layers and feature map nodes, enhancing the model’s sensitivity to objects of various sizes. CSFI advances this concept further by adding top-down and bottom-up cross-scale connections, thereby strengthening the bidirectional flow of high-level semantic information and low-level spatial information. Within each node, CSFI utilizes adaptive weighting and activation functions to dynamically adjust inter-feature relationships, thereby improving the model’s non-linear transformation capabilities. Furthermore, CSFI incorporates skip connections between intermediate layers to facilitate deeper feature fusion within the same hierarchy. This fusion process involves concatenating features, reparametrizing them using a RepBlock structure, and then adjusting the channel dimensions via a 1 × 1 convolution, thereby enhancing feature representation. The CSFI process integrates S2, S3, and F4, producing highly expressive encoder feature outputs.

### 2.4. Soft IoU-Aware

For object detection, we propose an innovative Soft IoU-Aware method designed to optimize the transformation from encoder outputs to final detection results. We recognize that directly using encoder outputs can lead to inconsistencies between classification scores and IoU values, which ultimately reduces detection accuracy.

The Soft IoU-Aware method addresses this issue by applying two parallel Multi-Layer Perceptrons (MLPs) to the encoder output features. The first MLP is dedicated to predicting the class scores, estimating the probability that a given candidate box belongs to a specific class. The second MLP is responsible for predicting the bounding box parameters and the corresponding IoU value.

Subsequently, we define a weighted loss function to further optimize the model, as represented by Formula (3):

(3) $$L_{total}=L_{cls}+\lambda_{1}L_{box}+\lambda_{2}L_{soft-IoU}$$

Here, $L_{cls}$ uses the cross-entropy loss function, and $L_{box}$ uses the Smooth L1 loss function. $\lambda_{1}$ and $\lambda_{2}$ are the weighting coefficients for $L_{box}$ and $L_{soft-IoU}$, respectively.

We define a soft-IoU loss function, as represented by Formula (4):

(4) $$L_{soft-IoU}=\frac{1}{N_{pos}}\sum_{i\epsilon pos}^{N}BCE({IoU}_{tru}-{IoU}_{pred})$$

During the inference process, the scores are calculated using the following formula:

(5) $$L_{soft-IoU}=\frac{1}{N_{pos}}\sum_{i\epsilon pos}^{N}BCE({IoU}_{tru}-{IoU}_{pred})$$

The top K features corresponding to the highest-weighted scores are selected, and they are used as inputs for the Transformer decoder, ensuring that the model has both high classification scores and high IoU values.

### 2.5. HDownsample and HUpsample

In deep-learning model architectures, adjusting the resolution of feature maps is a key step for effective feature representation. Although simple sampling operations, such as max pooling or convolution, can efficiently reduce the spatial resolution of feature maps, they risk omitting critical details that are essential for model performance. Additionally, upsampling methods—commonly utilizing nearest-neighbor interpolation or transposed convolution (deconvolution)—often introduce blurriness, adversely affecting the clarity and precision of feature details.

To overcome these limitations, we propose a novel composite sampling algorithm designed to maximize detail preservation while adjusting feature map resolution, thereby enhancing model precision. The details of our proposed downsampling and upsampling techniques are given below.

#### 2.5.1. HDownsample

**Figure 3:**
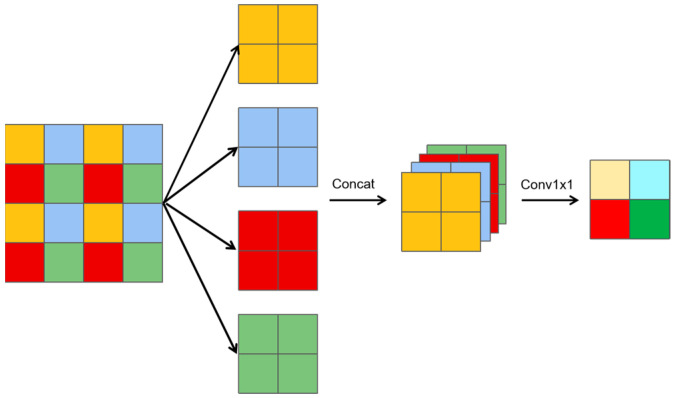
Slice downsampling.

As illustrated in Figure 3, a slice downsampling algorithm is introduced, which can be described in three key steps.

1. Slicing operation: The feature map is divided into multiple smaller slices, where each slice is half the size of the original feature map.
2. Channel concatenation: These smaller slices are concatenated along the channel dimensions, resulting in a feature map with four times the number of channels compared to the original.
3. 1 × 1 convolution: The concatenated feature map then undergoes a 1 × 1 convolution to reduce the number of channels back to the original count.

Let the original input feature be X. The downsampling operations yield three distinct features:

- A slice-downsampling feature (Y_dslice_), derived from the slicing and concatenation process;
- A max-pooling feature (Y_maxpool_), derived via max pooling with a 2 × 2 kernel and a stride of 2;
- A 3 × 3 convolutional feature (Y_conv3x3_), derived via a 3 × 3 convolution with a stride of 2.

The features Y_dslice_, Y_maxpool_, and Y_conv3×3_ are concatenated along the channel dimension. A 1 × 1 convolution is then applied to reduce the number of channels back to the original number of channels in feature X, resulting in the final output feature, which represents the fused feature map.

This series of composite downsampling operations is termed HDownsample, as represented in Formula (6), which optimizes the resolution of feature maps while enhancing their detail-capturing ability. By preserving crucial details, HDownsample ensures model precision and reliability.

(6) $$Y_{d}=Conv1\times 1(Concat(Y_{dslice},Y_{maxpool},Y_{conv3\times 3}))$$

#### 2.5.2. HUpsample

**Figure 4:**
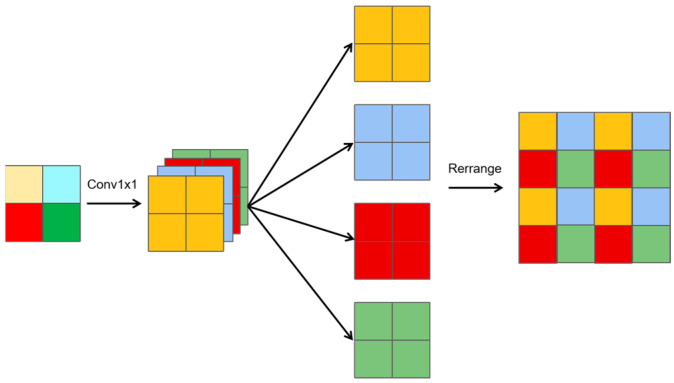
Slice upsampling.

Similarly, a slice-upsampling method is proposed, as shown in Figure 4, and can be summarized as follows:

1. 1 × 1 convolution: A 1 × 1 convolution is applied to generate features with four times the number of channels compared to the input.
2. Channel splitting: The resulting feature map is divided into four groups, with each group having the same number of channels as the original feature map.
3. Rearrangement: The four groups are mapped onto adjacent regions, creating a feature map with doubled height and width and the same number of channels as the original.

Let X represent the original input feature map. The upsampling process yields three distinct features:

- A slice-upsampling feature (Y_uslice_), derived from the slicing and rearrangement process;
- A nearest-neighbor feature (Y_nearest_), obtained using nearest-neighbor interpolation;
- A 3 × 3 deconvolution feature (Y_deconv3×3_), generated using a 3 × 3 deconvolution (transposed convolution) with a stride of 2.

The feature maps Y_uslice_, Y_nearest_, and Y_deconv3×3_ are concatenated along the channel dimension. A 1 × 1 convolution is then applied to reduce the number of channels back to the original number of channels in the feature X, resulting in the final output feature, which represents the fused feature map.

This series of composite upsampling operations is termed HUpsample, as represented by Formula (7). It optimizes the resolution of feature maps while enhancing their detail-capturing ability. By preserving crucial details, HUpsample ensures model precision and reliability.

(7) $$Y_{u}=Conv1x1(Concat(Y_{uslice},Y_{nearest},Y_{deconv3x3}))$$

### 2.6. SIoU Loss Function

IoU (Intersection over Union) [13] is a commonly used metric for evaluating object detection, measuring the overlap between the predicted and ground-truth boxes. However, IoU has limitations: when the boxes do not intersect, IoU cannot reflect the distance between them, making the loss function non-differentiable. Also, when the boxes are of the same size, IoU remains unchanged, making it difficult to differentiate the overlap.

To address these issues, several IoU variants have been proposed, such as GIoU [14], DIoU [15], CioU [15], EIoU [16], and SIoU [17]. GIoU solves the zero-gradient issue by using the minimum enclosing rectangle, but it degenerates to IoU when the boxes fully overlap. DIoU adds a Euclidean distance term for the box centers, helping to adjust the bounding box even with overlap. CIoU further adds an aspect ratio penalty, but it has limitations when the aspect ratio is the same. EIoU improves CIoU by separating aspect ratio and size influences and using a focal mechanism. SIoU provides a more accurate similarity measure by considering vector angles, width, height, and area differences. In this study, we adopt SIoU as the IoU loss metric. It consists of four parts: Angle Cost, Distance Cost, Shape Cost, and IoU Cost. The formula for calculating SIoU is as follows:

1. Angle Cost

**Figure 5:**
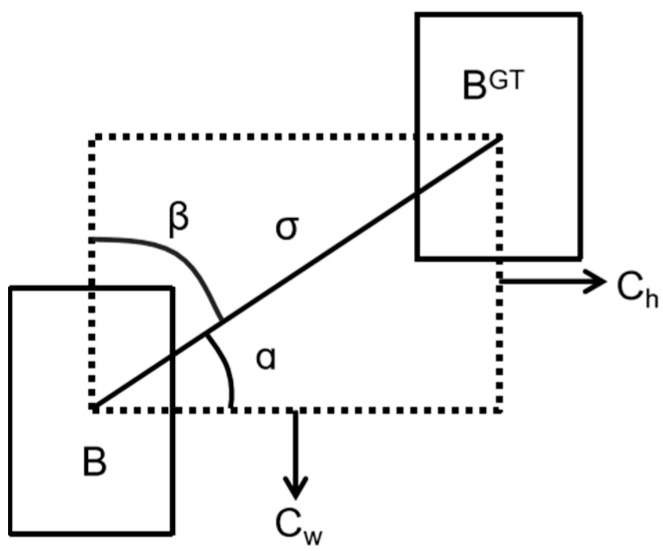
The scheme for calculating angle cost.

Figure 5 shows the contribution of calculation angle cost in the loss function. The formula is as follows:

(8) $$\Lambda =1-{sin}^{2}(arcsinx-\frac{\pi }{4})$$

where $x=\frac{c_{h}}{\sigma }=sin(\alpha )$, $\sigma =\sqrt{{\left(b_{c_{x}}^{gt}-b_{c_{x}}\right)}^{2}+{\left(b_{c_{y}}^{gt}-b_{c_{y}}\right)}^{2}}$, and $ch=max(b_{c_{y}}^{gt}-b_{c_{y}})-min(b_{c_{y}}^{gt}-b_{c_{y}})$.

2. Distance Cost

We introduce a parameter, γ, that increases with the angle, assigning a dynamic priority value to the distance cost. The formula is as follows:

(9) $$\Delta =\sum_{t=x,y}\left(1-e^{-\gamma pt}\right)$$

where $\rho_{x}={\left(\frac{b_{c_{x}}^{gt}-b_{c_{x}}}{c_{w}}\right)}^{2}$, $\rho_{y}={\left(\frac{b_{c_{y}}^{gt}-b_{c_{y}}}{c_{h}}\right)}^{2}$, γ=2−Λ.

3. Shape Cost

The shape loss formula is as follows:

(10) $$\Omega =\sum_{t=w,h}{\left(1-e^{-\omega_{t}}\right)}^{\theta }$$

where $\omega_{w}=\frac{\left|w-w^{gt}\right|}{max(w,w^{gt})},\omega_{h}=\frac{\left|h-h^{gt}\right|}{max(h,h^{gt})}$.

The parameter θ determines the scale of the shape cost in each dataset, and its value is crucial for the model’s performance. To prevent overemphasis on shape loss and reduce the movement of the predicted box, θ is set to 4.

4. IoU Cost

The IoU Cost is the ratio of the intersection to the union of the predicted and ground-truth boxes. The IoU loss formula is as follows:

(11) $$IoU=\left|\frac{B\cap B^{GT}}{B\cup B^{GT}}\right|$$

Finally, the calculation formula for the SIoU loss function is shown in the following formula:

(12) $$L_{box}=1-IoU+\frac{∆+\Omega }{2}$$

## 3. Results and Discussion

### 3.1. Datasets

**Figure 6:**
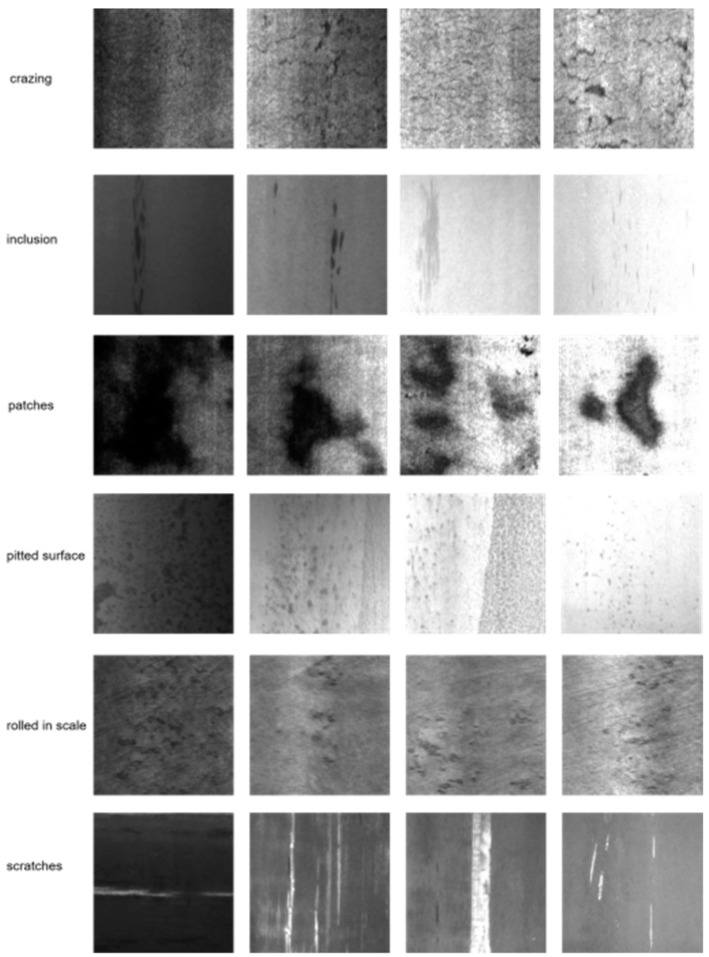
Examples from the NEU-DET steel surface defect dataset.

The NEU-DET dataset [18] is a collection focused on steel material defect detection, covering six different types of surface defects, namely, crazing, patches, inclusion, pitted surface, rolled-in scale, and scratches, with example images shown in Figure 6. The dataset contains a total of 1800 images. In this study, we split the dataset into 80% for training and 20% for testing, resulting in 1448 training images and 352 testing images.

**Figure 7:**
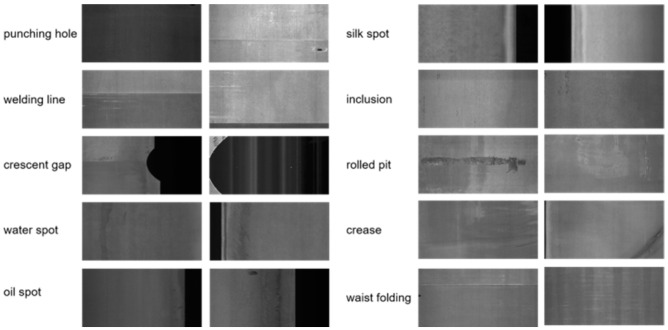
Examples from the GC10-DET steel surface defect dataset.

The GC10-DET dataset [19] is another dataset collected from real industrial environments, specifically corresponding to surface defect recognition. It contains ten types of surface defects as shown in Figure 7: punching holes, weld lines, crescent gaps, water spots, oil spots, silk spots, inclusions, rolled pits, creases, and waist folding. This dataset consists of 3570 grayscale images, which are also split into training and testing sets in an 8:2 ratio, containing 2856 training images and 714 testing images.

### 3.2. Experimental Setup

The experiments were conducted using an Ubuntu 18.04 operating system with eight Tesla V100 GPUs, utilizing CUDA 11.8 for acceleration. The deep-learning framework used was PaddlePaddle 2.6, and the programming language employed was Python 3.12.7. The training parameters were as follows: image input size, 640 × 640; TopK selection, 100; initial learning rate, 0.0001; number of epochs, 500; and batch size, 2.

To evaluate model performance, we employed mean average precision (mAP) as the primary metric, computed at three different IoU thresholds:

- mAP@0.5: The average precision at an IoU = 0.5, commonly used for object detection tasks.
- mAP@0.75: The average precision at a stricter IoU = 0.75, evaluating the model’s performance under higher precision requirements.

The mAP is calculated as follows:

(13) $$mAP=\frac{1}{K}\sum_{i=1}^{K}\;{\mathrm{AP}}_{i}$$

where AP denotes the average precision for each class, and K is the number of classes. The AP for each class is computed as follows:

(14) $$AP=\int_{0}^{1}\;P(R)dR$$

where P(R) is the precision as a function of recall, calculated as follows:

(15) $$P=\frac{TP}{TP+FP},R=\frac{TP}{TP+FN}$$

These metrics enable a comprehensive evaluation of the model’s detection accuracy at various levels of IoU, providing a more detailed performance assessment.

### 3.3. Results Obtained Using the Proposed Method

As shown in Table 2, the HCT-Det model demonstrated robust performance on both the NEU-DET and GC10-DET datasets, achieving mAP@0.5 values of 0.795 on the NEU-DET dataset and 0.733 on the GC10-DET dataset. These results indicate that the model is highly effective in detecting steel surface defects of varying scales and complexities.

**Figure 8:**
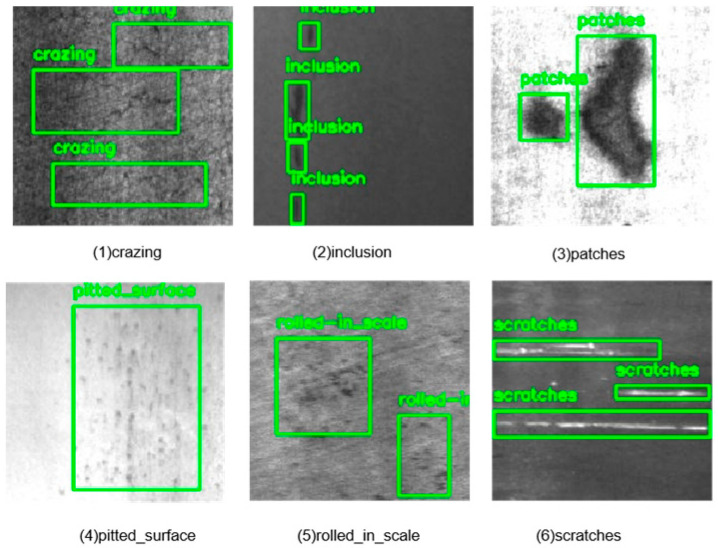
Detection results obtained using the NEU-DET dataset.

**Figure 9:**
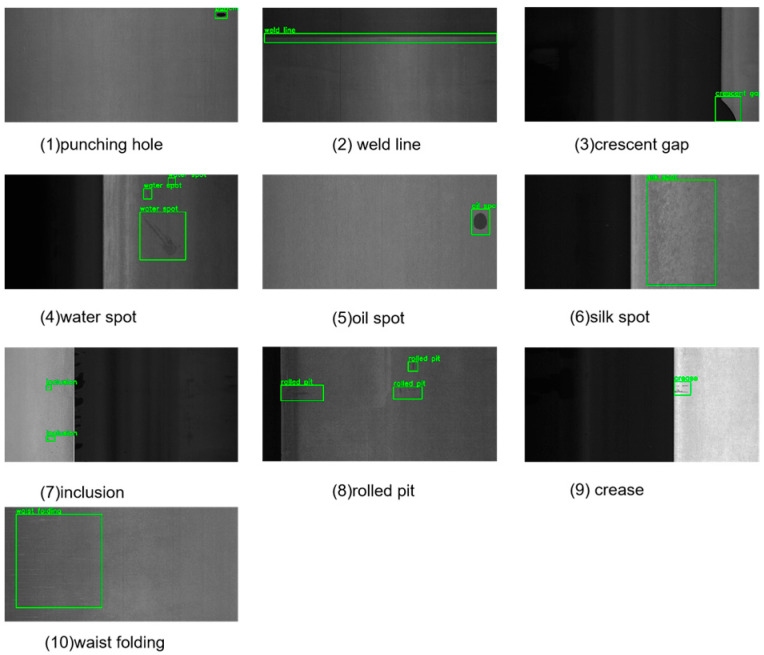
Detection results obtained using the GC10-DET dataset.

The visual results in Figure 8 and Figure 9 allow a qualitative assessment of the model’s performance. The images show that the HCT-Det model is capable of accurately detecting and localizing a variety of defects, ranging from small-scale features like crazing and inclusions to larger defects like rolled-in scales and waist folding. The model’s ability to handle diverse defect types and varying image complexities underscores its versatility and robustness.

### 3.4. Comparison with the State-of-the-Art Methods

To evaluate the performance of our proposed HCT-Det model, we conducted an experiment in which mAP@0.5 was used as the primary metric. To validate the effectiveness of our model, we compared it with several mainstream steel surface defect detection methods as baselines.

Before making any comparisons, we provide a brief overview of the following mainstream algorithms:

1. Faster RCNN [20] is a deep convolutional neural network integrated with Region Proposal Networks (RPNs) for the fast generation of object candidate regions and precise object detection.
2. SSD512 [21] is a single-shot multi-scale detection network that uses 512-pixel input images and can achieve fast and accurate detection of objects of various sizes through multi-scale feature maps.
3. YOLOv5 [22] is a single-shot multi-scale detection network that can quickly and accurately detect objects of various sizes using multi-scale feature maps. It is particularly effective for real-time object detection.
4. YOLOv8 [23] is the latest object detection model released by Ultralytics (Frederick, MD, USA) (it was made available in 2023); it introduces the C2f module, a decoupled head structure, and an anchor-free mechanism allowing faster detection and higher accuracy.
5. MSFT-YOLO [24] is a network based on YOLOv5 that integrates Transformer and BiFPN structures to improve detection capabilities and speed.
6. CABF-FCOS [25] is a fully convolutional single-stage network that incorporates channel attention mechanisms and bidirectional feature fusion. It is suitable for large-scale object detection.
7. ES-Net [26] is a highly efficient scale-aware network for detecting small defects. It significantly improves detection accuracy with respect to small defects.
8. DEA_RetinaNet [27] is a deep neural network based on channel attention mechanisms that optimizes the RetinaNet architecture.
9. DCC-CenterNet [28] is an anchor-free convolutional neural network that utilizes a center-point detection mechanism for object localization.
10. MSC-Dnet [29] is a multi-scale defect detection network based on an improved version of Faster RCNN, aimed at enhancing the detection of defects at different scales.

As shown in Table 3, compared with mainstream object detection models (Faster RCNN, SSD512, YOLOv5, and YOLOv8), our proposed HCT-Det achieved higher detection accuracy on two public datasets. This strongly demonstrates the effectiveness of our proposed model, especially the Window self-attention Transformer and ResNet fusion module (WSA-R), Intra-Scale Feature Interaction (ISFI), Cross-Scale Feature Interaction (CSFI), and Soft-IoU Aware. The WSA-R block reduced computational load while also strengthening local feature connections through parallel residual convolutional blocks, avoiding local feature loss due to window limitations. ISFI enhanced the model’s ability to recognize small targets. CSFI facilitated the thorough fusion of features. Soft IoU-Aware ensured the model had high classification scores and high IoU, reducing false-positive errors. The HDownsample downsampling and HUpsample upsampling modules further reduced feature loss in operations. At the same time, the performance of models such as MSFT-YOLO, CABF-FCOS, and DEA_RetinaNet showed that introducing attention mechanisms and global feature extraction could significantly improve model accuracy. Models like ES-Net and MSC-Dnet, by extracting multi-scale features, adapt to different defect sizes, thereby enhancing the recognition rate for minor defects and further improving detection accuracy.

### 3.5. Comparison with Transformer Methods

To further evaluate the characteristics of HCT-Det in terms of parameter count and computational efficiency among Transformer methods, we compared it with two other advanced Transformer methods:

1. MVitV2 [30] is an improved Vision Transformer that uses hierarchical encoders for multi-scale feature extraction, enhancing the handling of both local and global image information. It performs well in complex scenes with objects at various scales.
2. VitDet [31] is an object detection framework based on a Vision Transformer, incorporating Transformer encoder and decoder structures to improve detection accuracy by effectively handling long-range dependencies in images.

As shown in Table 4, the HCT-Det model significantly outperforms MVitV2 and VitDet in terms of parameter count and FLOPs. Specifically, HCT-Det has 162 million parameters and 0.7 trillion FLOPs, much lower than MVitV2’s 238 million parameters and 1.3 trillion FLOPs and VitDet’s 331 million parameters and 1.9 trillion FLOPs. This indicates that HCT-Det has a substantial advantage in computational efficiency, especially when processing high-resolution images. Additionally, HCT-Det performs well in regard to the more stringent mAP@0.75 metrics, further proving its superiority in detecting small targets and handling complex backgrounds.

### 3.6. Effects of Using Different IoU Loss Functions

To evaluate the impact of various IoU loss functions on the performance of our HCT-Det model, we conducted experiments in which we tested the effects of applying GIoU, DIoU, CIoU, EIoU, and SIoU on the model’s performance on the NEU-DET and GC10-DET datasets. The results are detailed in Table 5.

Table 5 details the performance of different IoU loss functions with respect to the NEU-DET and GC10-DET datasets. The data reveal that while the IoU loss functions exhibited closely matched performance, the SIoU loss function outperformed the others, showing a slight but noticeable improvement on both datasets. This edge can be attributed to SIoU’s comprehensive consideration of geometric factors during loss calculation, which more effectively guides a model towards accurate target localization. The consistent outperformance of SIoU across both datasets suggests that incorporating a richer set of geometric insights into loss functions can lead to enhanced detection accuracy. This insight is valuable for the development of future object detection models, indicating that the choice of loss function—aiming for one that more fully reflects the relationship between predicted and ground-truth boxes—is pivotal for achieving superior detection performance.

### 3.7. Effects of Using Different Numbers of WSA-R Fusion Blocks

**Figure 10:**
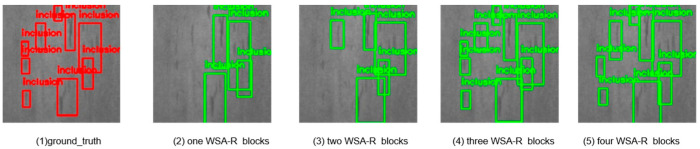
Detection results obtained when employing different numbers of WSA-R blocks on the NEU-DET dataset.

**Figure 11:**
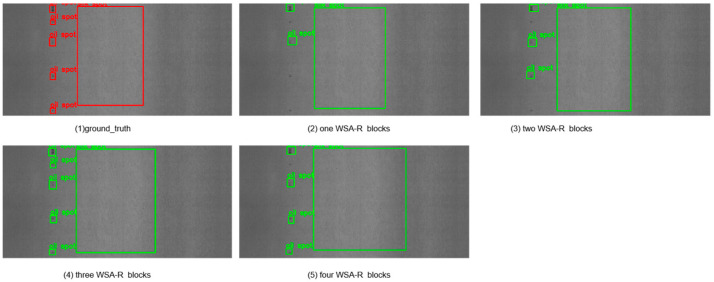
Detection results obtained when employing different numbers of WSA-R blocks on the GC10-DET dataset.

To evaluate the impact of using different numbers of WSA-R fusion blocks on detection accuracy, we conducted experiments with varying configurations. The results are summarized in Table 6 and visually depicted in Figure 10 and Figure 11.

Configuration Details:

- Numbers = 1: The WSA-R block outputs S1, which is downsampled and passed through a 1 × 1 convolution to produce S2. Similarly, S2 is downsampled and passed through a 1 × 1 convolution to produce S3.
- Numbers = 2: The first WSA-R block outputs S1, and the second WSA-R block outputs S2. S2 is then downsampled and passed through a 1 × 1 convolution to produce S3.
- Numbers = 3: The first WSA-R block outputs S1, the second WSA-R block outputs S2, and the third WSA-R block outputs S3.
- Numbers = 4: The second WSA-R block outputs S2, the third WSA-R block outputs S3, and the fourth WSA-R block outputs S4.

The data reveal that the model’s detection accuracy initially improved with an increasing number of WSA-R blocks but then decreased after reaching an optimal point. Specifically, the highest detection accuracy on both datasets was achieved with three WSA-R fusion blocks, indicating that this configuration strikes the best balance between effective feature fusion and computational efficiency.

The visual results in Figure 10 and Figure 11 allow a qualitative assessment of the model’s performance. The images show that as the number of WSA-R blocks increases from one to three, the detection of small targets improves significantly. This indicates that the additional blocks enhance the model’s ability to capture and localize small targets more effectively. However, when the number of blocks increases to four, the ability to detect small targets decreases, suggesting that too many blocks may introduce redundant information and hinder the model’s ability to capture small targets effectively.

### 3.8. Effect of Using Soft IoU-Aware Method

To evaluate the impact of the Soft IoU-Aware method on the detection accuracy of our model, we conducted experiments with and without this mechanism. The results are summarized in Table 7 and provide a clear indication of the method’s effectiveness.

The application of the Soft IoU-Aware method significantly improved the model’s detection accuracy in regard to both the NEU-DET and GC10-DET datasets. As shown in Table 7, without the Soft IoU-Aware mechanism, the model achieved mAP@0.5 scores of 0.779 on the NEU-DET dataset and 0.721 on the GC10-DET dataset. After the Soft IoU-Aware mechanism was incorporated, the mAP@0.5 scores increased to 0.795 and 0.733, respectively. This improvement is also evident in the more stringent mAP@0.75 metrics, where the Soft IoU-Aware method further enhances the model’s localization ability.

The Soft IoU-Aware method introduces a soft loss constraint during training, ensuring alignment between high Intersection-over-Union (IoU) values and high classification scores. This alignment effectively reduces false-positive errors and improves the model’s ability to accurately localize objects, especially in complex scenes with overlapping or crowded objects.

### 3.9. Effects of Using Different Sampling Methods

#### 3.9.1. Comparison Between Different Downsampling Methods

To evaluate the impact of different downsampling methods on the model’s detection accuracy, we conducted experiments using three methods: max pooling, a 3 × 3 convolution with a stride of 2, and our proposed HDownsample method. The results are summarized in Table 8.

The results indicate that HDownsample achieved the highest accuracy on both the NEU-DET and GC10-DET datasets, respectively. This demonstrates that HDownsample is more effective at preserving key feature information than traditional methods like max pooling and 3 × 3 convolution with a stride of 2. The composite operations in HDownsample better retained the detailed information in the feature maps, thereby enhancing the model’s detection performance.

#### 3.9.2. Comparison Between Different Upsampling Methods

To evaluate the impact of different upsampling methods on the model’s detection accuracy, we conducted experiments using three methods: nearest-neighbor interpolation, 2 × 2 stride 3 × 3 transposed convolution, and our proposed HUpsample method. The results are summarized in Table 9.

The results demonstrate that HUpsample outperformed the other methods on both datasets. This indicates that HUpsample was more effective in preserving and enhancing the detailed features of the feature map. The composite operations in HUpsample allow for a more accurate recovery of feature map details, thus further improving the model’s detection performance.

## 4. Conclusions

In this paper, we propose a steel surface defect detection model, HCT-Det. We first designed the WSA-R block module, which reduces computation through a window-based self-attention mechanism (WSA Block) while also enhancing local feature connections using parallel residual convolution blocks (Res Blocks). This approach prevents the loss of local features due to window limitations. Next, in the encoder, we performed Intra-Scale Feature Interaction (ISFI) on the deepest features to enhance recognition of small targets. This was followed by Cross-Scale Feature Interaction (CSFI) with two other scales to fully fuse the features, thereby improving detection accuracy with respect to large, medium, and small targets. Third, we used Soft IoU-Aware, where soft loss constraints during training ensured that high-IoU features produce high classification scores, while low-IoU features produce low classification scores. This ensures the model had both high classification accuracy and high IoU. Additionally, we designed composite HDownsample and HUpsample modules to reduce feature loss during operations and further improve the model’s detection performance.

The experimental results show that our HCT-Det model achieved accuracies of 0.795 and 0.733 on the NEU-DET and GC10-DET datasets, respectively, outperforming traditional models. However, the HCT-Det model is still relatively large. Future research will focus on the design of lightweight models.

## Figures and Tables

**Table 1:** Comparison of parameters: MSA vs. WSA.

Parameter	MSA	WSA
Computational Complexity	Quadratic	Linear
Global Dependency	Global	Local
Scalability	Low	High

**Table 2:** Results obtained by applying the HCT-Det model to the NEU-DET and GC10-DET datasets.

Dataset	mAP@0.5	mAP@0.75
NEU-DET	0.795	0.550
GC10-DET	0.733	0.448

**Table 3:** Comparison with state-of-the-art methods.

Method	NEU-DET (mAP@0.5)	GC10-DET (mAP@0.5)
Faster RCNN	0.696	0.627
SSD512	0.723	0.659
Yolov5	0.682	0.673
Yolov8	0.757	0.683
MSFT-YOLO	0.752	-
CABF-FCOS	0.767	-
ES-Net	0.791	-
DEA_RetinaNet	0.791	-
DCC-CenterNet	0.794	0.619
MSC-Dnet	0.794	0.716
HCT-Det	0.795	0.733

**Table 4:** Comparison with Transformer methods.

Method	Params	Flops	NEU-DET		GC10-DET	
			mAP@0.5	mAP@0.75	mAP@0.5	mAP@0.75
MVitV2	238 M	1.3 T	0.729	0.478	0.697	0.415
VitDet	331 M	1.9 T	0.741	0.492	0.702	0.423
HCT-DET	162 M	0.7 T	0.795	0.550	0.733	0.448

**Table 5:** Comparison of different IoU loss functions.

Method	NEU-DET		GC10-DET	
	mAP@0.5	mAP@0.75	mAP@0.5	mAP@0.75
GIoU	0.781	0.525	0.718	0.422
DIoU	0.779	0.523	0.725	0.430
CIoU	0.783	0.528	0.726	0.432
EIoU	0.787	0.532	0.729	0.435
SIoU	0.795	0.550	0.733	0.448

**Table 6:** Comparison with different numbers of WSA-R blocks.

Numbers	NEU-DET		GC10-DET	
	mAP@0.5	mAP@0.75	mAP@0.5	mAP@0.75
1	0.782	0.526	0.715	0.418
2	0.790	0.541	0.723	0.430
3	0.795	0.550	0.733	0.448
4	0.793	0.548	0.730	0.445

**Table 7:** Comparison with and without Soft IoU-Aware.

Method	NEU-DET		GC10-DET	
	mAP@0.5	mAP@0.75	mAP@0.5	mAP@0.75
Without Soft-IoU aware	0.779	0.523	0.721	0.428
With Soft-IoU aware	0.795	0.550	0.733	0.448

**Table 8:** Comparison of different downsampling methods.

Method	NEU—DET		GC10—DET	
	mAP@0.5	mAP@0.75	mAP@0.5	mAP@0.75
Max Pooling	0.783	0.520	0.724	0.415
Conv3 × 3, s = 2	0.785	0.525	0.726	0.421
HDownsample	0.795	0.550	0.733	0.448

**Table 9:** Comparison of different upsampling methods.

Method	NEU—DET		GC10—DET	
	mAP@0.5	mAP@0.75	mAP@0.5	mAP@0.75
Nearest Neighbor	0.778	0.515	0.725	0.416
Deconv3 × 3, s = 2	0.786	0.528	0.729	0.425
HUpsample	0.795	0.550	0.733	0.448

## Data Availability

The original data presented in the study are openly available in the following publicly accessible repositories: (1) NEU-DET dataset: http://faculty.neu.edu.cn/songkechen/zh_CN/zdylm/263270/list/ (accessed on 11 June 2024). (2) GC10-DETdataset: https://github.com/lvxiaoming2019/GC10-DET-Metallic-Surface-Defect-Datasets (accessed on 11 June 2024).
